# Effect of 2-Hydroxyethyl Methacrylate on Antioxidant Responsive Element-Mediated Transcription: A Possible Indication of Its Cytotoxicity

**DOI:** 10.1371/journal.pone.0058907

**Published:** 2013-03-14

**Authors:** Ai Orimoto, Takahiro Suzuki, Atsuko Ueno, Tatsushi Kawai, Hiroshi Nakamura, Takao Kanamori

**Affiliations:** 1 Department of Endodontics, School of Dentistry, Aichi-Gakuin University, Nagoya, Japan; 2 Department of Biochemistry, School of Dentistry, Aichi-Gakuin University, Nagoya, Japan; 3 Department of Gerodontology, School of Dentistry, Aichi-Gakuin University, Nagoya, Japan; 4 Department of Dental Material Science, School of Dentistry, Aichi-Gakuin University, Nagoya, Japan; University of Texas Health Science Center, United States Of America

## Abstract

**Background:**

The resin monomer 2-hydroxyethyl methacrylate (HEMA) is known to be more cytotoxic than methyl methacrylate (MMA). Using a luciferase reporter assay system, we previously showed that MMA activates the glutathione S-transferase alpha 1 gene (*Gsta1*) promoter through the anti-oxidant responsive element (ARE). However, it is not known whether HEMA induces ARE-mediated transcription.

**Methodology/Principal Findings:**

We further developed the reporter system and studied the concentration-dependent effect of HEMA on ARE enhancer activity. The revised system employed HepG2 cells stably transfected with a destabilized luciferase reporter vector carrying 2 copies of the 41-bp ARE region of *Gsta1*. In this system, MMA increased ARE activity by 244-fold at 30 mM; HEMA augmented ARE activity at 3 mM more intensely than MMA (36-fold versus 11-fold) and was equipotent as MMA at 10 mM (56-fold activation); however, HEMA failed to increase ARE activity at 30 mM. In HepG2 cells, HEMA detectably lowered the cellular glutathione levels at 10 mM and cell viability at 30 mM, but MMA did not.

**Conclusions:**

These results suggest that the low-concentration effect of HEMA on ARE activity reflects its cytotoxicity. Our reporter system used to examine ARE activity may be useful for evaluating cytotoxicities of resin monomers at concentrations lower than those for which cell viabilities are reduced.

## Introduction

Acrylic resins have been used in the fields of clinical dentistry and medicine. However, unpolymerized resin monomers can be released into the surrounding tissues and organs before and even after polymerization [1], which may cause cytotoxic effects such as micronucleus formation [2], [3], oxidative stress [3], [4], and reduced cell viability [1]. Resin monomers can lead to a decrease in intracellular glutathione (GSH) levels through a detoxification process [1], [3], [5]. It is crucial to understand resin monomer detoxification responses and their relationship with cytotoxicity to evaluate the safety in the use of these dental materials.

Methyl methacrylate (MMA) is an acrylic resin monomer and one of the most widely used materials in dentistry. MMA is used at high concentrations to produce polymers for restorative dental materials because the median lethal dose (LD_50_) of MMA is reportedly very high (over 80 mM) compared with other acrylic resin monomers [2], [6]. On the other hand, 2-hydroxyethyl methacrylate (HEMA) is one of the most common components used in dentin-bonding systems due to its positive effect on bond strength [7]–[9]. HEMA is a water-soluble acrylic resin monomer and can easily diffuse throughout dentin tubules, which can reduce cell viability in the underlying odontoblastic cell layer [10]. In cultured cells such as human pulp fibroblasts and human gingival epithelial Smulow-Glickman cells, HEMA has been reported to reduce cell viability in a dose-dependent manner, as well as to induce GSH depletion, reactive oxygen species production, cell cycle perturbation, and apoptosis [5], [11]–[13]. Some studies showed that the LD_50_ value of HEMA was lower than that of MMA in various cells [1], [2], [6], [14].

We previously showed that MMA up-regulated gene expression of xenobiotic metabolizing phase II enzymes such as glutathione S-transferases (GSTs) [15], [16]. Phase II enzymes function as intracellular detoxification systems of toxic compounds; GSTs are a family of enzymes that catalyze the conjugation of electrophilic compounds with GSH. Antioxidant and electrophilic reagents enhance the transcription of phase II enzymes through the antioxidant responsive element (ARE) present in the 5′-flanking regions of their genes [17], [18]. The central transcription factor involved in ARE-mediated gene expression is nuclear factor erythroid 2-related factor 2 (Nrf2), which is retained under basal conditions by a cytosolic repressor protein, Kelch-like ECH-associated protein 1 (Keap1) [18]–[21]. Keap1 possesses highly reactive sulfhydryl groups and is a cellular sensor for electrophiles and oxidants [22]. Modification of these sulfhydryl groups induces conformational changes in Keap1, disruption of the Nrf2-Keap1 complex [23], release of Nrf2 from Keap1, accumulation of Nrf2 in nucleus [24]–[27], and activation of the ARE [21], [28], [29]. It is likely that two types of pathways are involved in the keap1 modification caused by resin monomers and other electrophiles [22]: (1) disulfide-bond formation between reactive cysteine residues on Keap1 exposed to oxidative stress following depletion of GSH resulting from its conjugation with resin monomers, and (2) direct conjugation of reactive cysteine residues on Keap1 with resin monomers.

In our previous study, we focused on the mechanism of MMA-induced expression of *Gsta1* and showed that MMA increased *Gsta1* promoter activity through ARE by using a luciferase reporter assay [16]. However, it is not known whether other resin monomers induce ARE-mediated transcription. As described above, ARE activity is a key component in response to toxic xenobiotics and can be used as a sensitive index of dose-dependent cytotoxicity of resin monomers. In the present study, we tested the hypothesis that HEMA, which is probably more toxic than MMA, induced ARE-mediated transcription at lower concentrations than MMA. We developed our previous ARE-luciferase reporter assay system, examined the dose effects of MMA and HEMA on ARE enhancer activity, and studied theirs relation to intracellular GSH levels and cell viability.

## Results

### Improvement of the ARE-luciferase reporter vector

We previously showed that MMA increased *Gsta1* promoter activity by 2.6-fold at 10 mM in HepG2 cells using a luciferase reporter vector (pGL4.10-*GSTa1*pro990) containing the 5′-flanking region (-990 to +46 bp) of *Gsta1* with the 41-bp ARE region (-729 to -689 bp; 2 ARE consensus sequences are included) [16]. We also reported that MMA increased ARE activity by 2.8-fold at 10 mM using a reporter vector (pTAL-2E-Luc) containing 2 tandem copies of the *Gsta1*-derived ARE region upstream of a TATA-like promoter [16]. To further study and compare the dose effects of resin monomers on ARE activity, we attempted to engineer ARE-luciferase reporter vectors that would enhance the MMA-induced activation rate of ARE activity.

**Figure 1:**
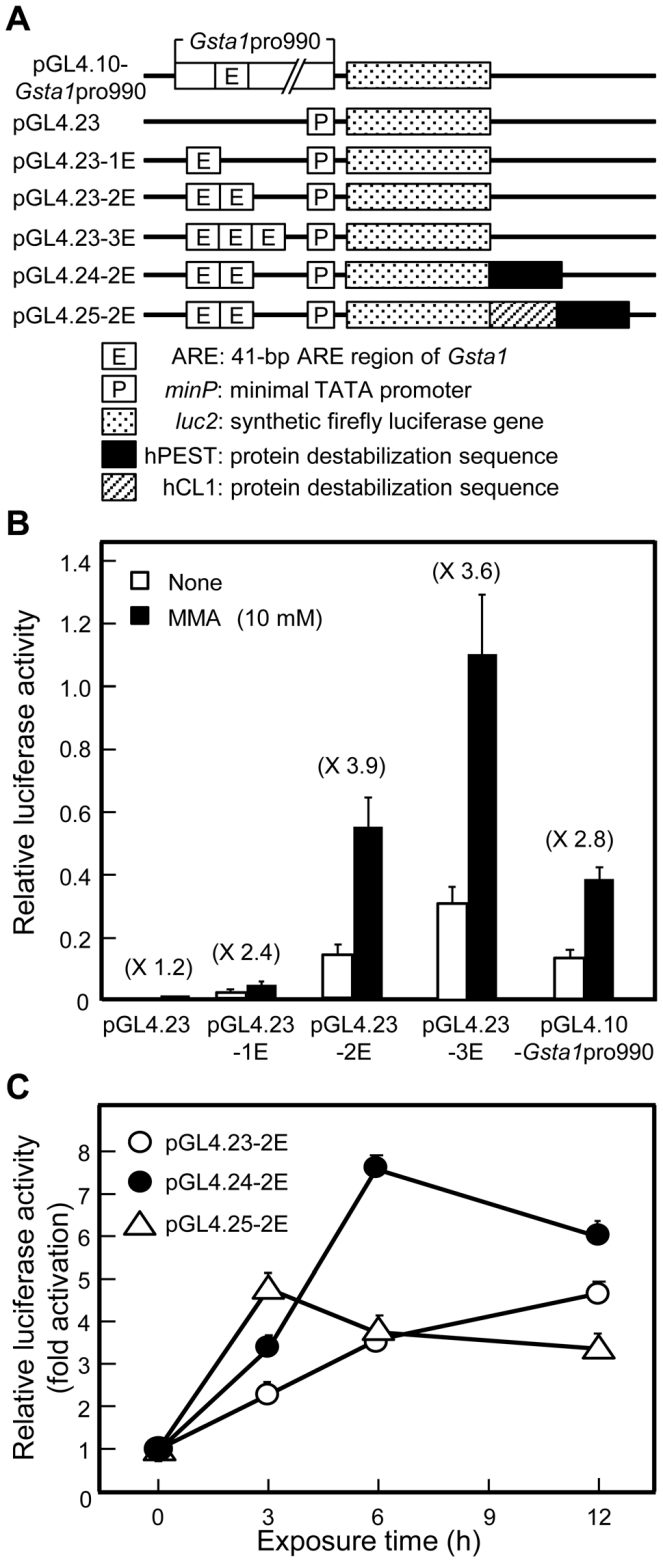
Effect of MMA on luciferase reporter activity in HepG2 cells transfected with vectors containing *Gsta1*-ARE. (A) Structures of reporter constructs. The vector pGL4.10-*Gsta1*pro990 contains 5′-flanking region (-990 to +46; the 41-bp of ARE region is located -729 to -689) immediately upstream of the synthetic firefly luciferase reporter gene (*luc2*), which was previously constructed [16]. The 41-bp of ARE region contains 2 ARE consensus sequences (see “Materials and Methods”). The vector pGL4.23 is a plasmid with a minimal promoter (*minP*), which contains a TATA-box promoter element immediately upstream of *luc2* and immediately downstream of the multiple cloning sites. The vectors pGL4.23-1E, pGL4.23-2E, and pGL4.23-3E contain 1, 2, and 3 copies, respectively, of the *Gsta1*-derived ARE upstream of *minP*. The vectors pGL4.24-2E and pGL4.25-2E contain 2 copies of the *Gsta1*-ARE and *minP* (“ARE-ARE-minP unit”) immediately upstream of *luc2* with destabilization sequences hPEST and hCL1-hPEST, respectively. (B) Effects of MMA on the promoter activity of *minP* downstream of the ARE(s). HepG2 cells were co-transfected with phRL-CMV and 1 of the 5 plasmid vectors (pGL4.23, pGL4.23-1E, pGL4.23-2E, pGL4.23-3E, and pGL4.10-GSTa1pro990), cultured for 24 h, incubated without or with MMA (initial concentration, 10 mM) for 6 h, and subjected to the assay for firefly and *Renilla* luciferase activities. Firefly luciferase activities were normalized to *Renilla* luciferase activities. Data are presented as the mean ± SD (n  =  4–21). (C) Increase in response rate of ARE-reporter activity using the destabilized luciferase gene. Cells were co-transfected with phRL-CMV and 1 of 3 vectors (pGL4.23-2E, pGL4.24-2E, and pGL4.25-2E), cultured for 24 h, incubated with MMA (initial concentration, 10 mM) for the indicated periods, and subjected to the assay for firefly and *Renilla* luciferase activities. Firefly luciferase activities were normalized to *Renilla* luciferase activities; for each vector, relative luciferase activities were expressed with the control value, obtained without exposure to MMA (1.342 for pGL4.23-2E, 0.203 for pGL4.24-2E, and 0.026 for pGL4.25-2E; the decrease of basal luciferase activity was dependent on protein destablization sequences), taken as 1.0. Data are shown as the mean ± SD (n  =  4–6).

We used the luciferase reporter vector pGL4.23 because the pGL4 vector family has been designed to increase the reliability of reporter gene expression by eliminating transcription factor binding sites in the vector sequence. First, we constructed luciferase reporter vectors containing 1, 2, or 3 tandem copies of the *Gsta1*-ARE upstream of a minimal promoter (*minP*) and a synthetic firefly luciferase gene (*luc2*), and named them pGL4.23-1E, -2E, and -3E, respectively (Figure 1A). When HepG2 cells were transfected with each of the 3 vectors, and incubated for 6 h without or with MMA (initial concentration, 10 mM; we studied the effect of transient exposure of cells to acrylic monomers. See “Materials and Methods”), MMA enhanced expression of the reporter luciferase by 2.4-fold, 3.9-fold, and 3.6-fold, respectively (Figure 1B); the activation rate was maximum in the cells transfected with pGL4.23-2E, although basal luciferase activity was the highest in the cells transfected with pGL4.23-3E. Thus, we chose 2 copies of *Gsta1*-ARE for further construction of the reporter gene.

We next examined the destabilized luciferase genes containing protein degradation sequences, which are known to increase the response rate of the reporter genes. The vectors pGL4.24-2E and pGL4.25-2E constructed by subcloning 2 copies of the *Gsta1*-ARE into pGL4.24 and pGL4.25 vectors, respectively, containing the protein destabilization sequences hPEST and hCL1-hPEST, respectively (Figure 1A). In HepG2 cells transfected with pGL4.24-2E, reporter activity reached a maximum (8-fold activation) at 6 h after exposure to 10 mM MMA (Figure 1C). As shown in Figure 1C, HepG2 cells transfected with pGL4.24-2E were more responsive to MMA than those transfected with pGL4.23-2E or pGL4.25-2E.

### HEMA increased ARE-mediated promoter activity at lower concentrations than MMA

**Figure 2:**
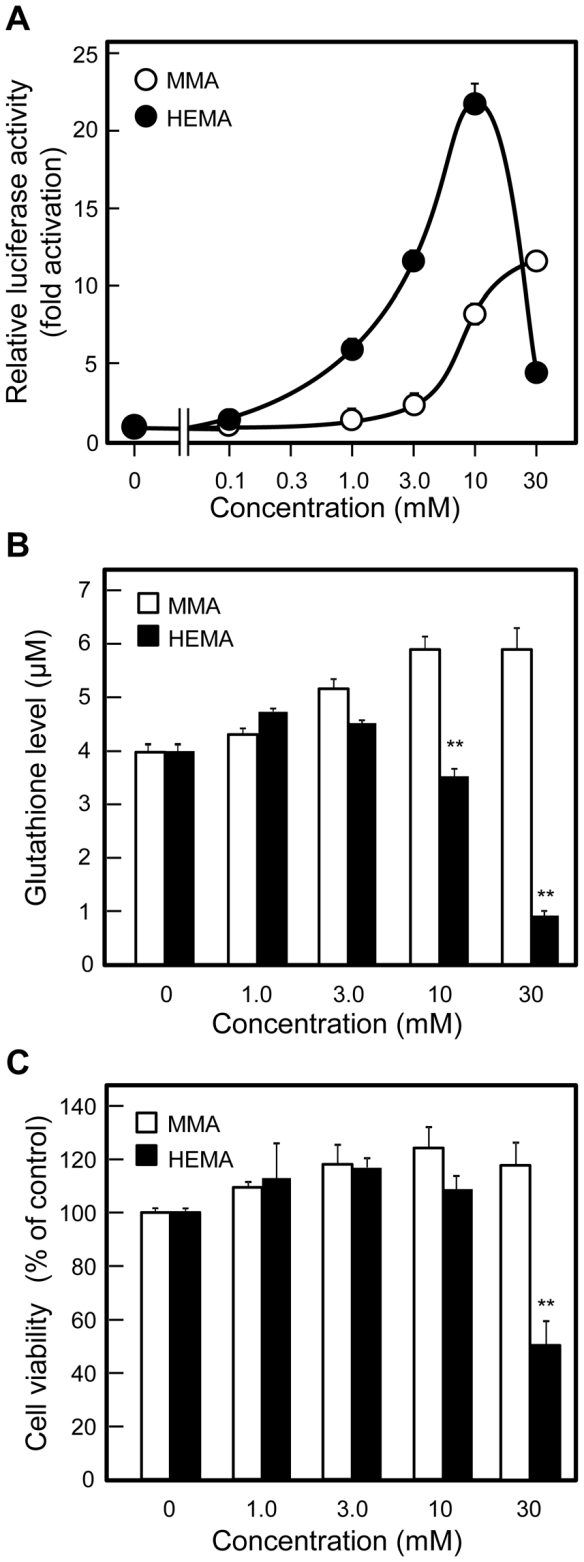
Concentration-dependent effect of MMA and HEMA on ARE activity, intracellular GSH levels, and cell viability. (A) Effect on promoter activity in HepG2 cells transfected with the vector pGL4.24-2E. HepG2 cells were co-transfected with pGL4.24-2E and phRL-CMV, cultured for 24 h, incubated for 6 h with indicated initial concentrations of MMA or HEMA, and subjected to the assay for the luciferase activities. Relative luciferase activities were expressed with the control value, obtained after incubation without exposure to MMA or HEMA, taken as 1.0. Data are presented as the mean ± SD (n  =  4–6). (B) Effect on intracellular GSH levels. Cells were incubated for 6 h with indicated initial concentrations of MMA or HEMA, and subjected to the assay for intracellular levels of GSH. Data are presented as the mean ± SD (n  =  3). (C) Effect on cell viability. Cells were incubated for 24 h with indicated initial concentrations of MMA or HEMA, and subjected to the assay for cell viability using WST-8. Cell viability is expressed with the control value, obtained after incubation without exposure to MMA or HEMA, taken as 100%. Data are presented as the mean ± SD (n  =  3). ^*^ *p* < 0.05, ^**^ *p* < 0.01.

When HepG2 cells were transfected with pGL4.24-2E and incubated without additives or with various initial concentrations of MMA or HEMA for 6 h, HEMA increased promoter activity at lower concentrations, unlike MMA (Figure 2A). At 1, 3, and 10 mM, HEMA increased the promoter activity by 5.6-, 11-, and 22-fold, respectively, whereas MMA increased it by 1.3-, 2.4-, and 8.3-fold, respectively (Figure 2A). At 30 mM, MMA further enhanced the promoter activity up to 12-fold, whereas HEMA less effectively increased it up to 4.7-fold (Figure 2A).

Based on the microscopic inspection of cells after the 6-h incubation, we concluded that MMA and HEMA at concentrations under 30 mM did not exert observable effects on the cell number and the shape of HepG2 cells; however, HEMA significantly reduced both *Renilla* luciferase activity derived from the internal control vector phRL-CMV (cytomegalovirus) and firefly luciferase activity derived from pGL4.24-2E at the concentration of 30 mM, suggesting a nonspecific inhibition of transcription activity by HEMA at 30 mM. To confirm this observation, we also examined the effects of 30 mM HEMA on SV40 (simian virus 40), TK (herpes simplex virus thymidine kinase), and CMV promoter activities; HepG2 cells transfected with a vector carrying each of the 3 promoters in a single dish were divided into small wells and subjected to treatment with 30 mM HEMA and the luciferase assay (Figure S1). Under these experimental conditions, HEMA at 30 mM significantly reduced all the promoter activities tested after 6 h of incubation (Figure S1C), indicating its nonspecific inhibition of transcription.

### Reduction of cellular GSH levels and cell viability at higher concentrations of HEMA in HepG2 cells

When HepG2 cells were incubated for 6 h with several initial concentrations (0–30 mM) of MMA or HEMA, MMA increased intracellular GSH levels in a dose-dependent manner (Figure 2B). In contrast, HEMA lowered GSH levels at 10 and 30 mM, although 1 and 3 mM HEMA increased GSH levels. Cell viability was lowered by 30 mM HEMA after 24-h incubation (Figure 2C), but not after 6-h incubation (data not shown).

### HEMA-induced ARE activation via the Keap1-Nrf2 pathway

**Figure 3:**
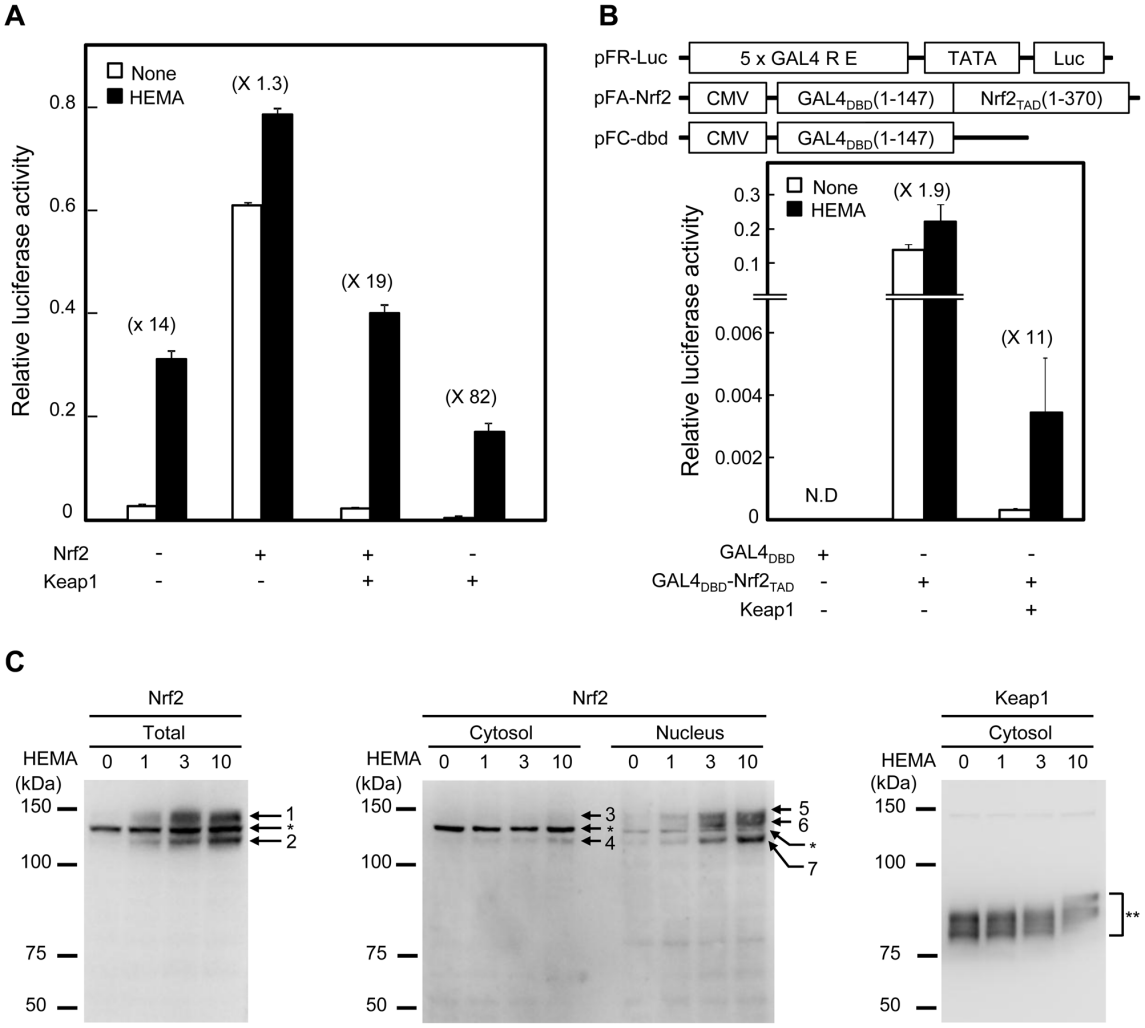
Effect of HEMA on the Keap1-Nrf2 pathway. (A) Effect of overexpression of Keap1 and Nrf2 on HEMA-induced ARE activation. HepG2 cells were transfected with pFlag-CMV2-hNrf2 and/or pCMV-SPORT6/hKeap1in addition to the pGL4.24-2E ARE-luciferase vector; pcDNA3 plasmid was used as a control vector. The vector phRL-CMV was also co-transfected into cells as an internal control for normalizing the reporter luciferase activity. The transfected cells were cultured for 24 h, incubated without or with HEMA (3 mM) for 6 h, and subjected to the assay for firefly and *Renilla* luciferase activities. Firefly luciferase activities were normalized to *Renilla* luciferase activities. Data are presented as the mean ± SD (n  =  3). +, expressed; -, non-expressed. (B) Transactivation activity of Nrf2 determined by a GAL4-luciferase reporter assay. Plasmid vectors used for the GAL4 assay were as follows: pFR-Luc, a vector containing 5 copies of GAL4-responsive elements (5 x GAL4-RE) and a TATA-box promoter element immediately upstream of the firefly luciferase gene (Luc); pFA-Nrf2, a vector expressing for the fusion protein GAL4_DBD_-Nrf2_TAD_ consisting of the DNA binding domain of the yeast GAL4 transcription factor (GAL4_DBD_) and the transactivation domain of the human Nrf2 (Nrf2_TAD_); pFC-dbd, a vector expressing GAL4_DBD_ only. HepG2 cells were co-transfected with pFA-Nrf2 and/or pCMV-SPORT6/hKeap1 in addition to pFR-Luc. As negative controls for pFA-Nrf2 and pCMV-SPORT6/hKeap1, pFC-dbd and pcDNA3, respectively, were used. The vector phRL-CMV was also co-transfected into cells (see above). The transfected cells were cultured for 24 h, incubated without or with HEMA (3 mM) for 6 h, and subjected to the assay for firefly and *Renilla* luciferase activities. Firefly luciferase activities were normalized to *Renilla* luciferase activities. Data are presented as the mean ± SD (n  =  3). +, expressed; -, non-expressed. (C) Western blot analysis of Nrf2 and Keap1 in HEMA-treated HepG2 cells. HepG2 cells were incubated without or with HEMA (1, 3, and 10 mM) for 6 h, and proteins in total cell lysates and in nuclear and cytosolic extracts were separated by SDS-PAGE followed by western blot analysis using anti-Nrf2 and anti-Keap1 antibodies. Arrows 1-7 indicate Nrf2. Bands marked by * are most probably nonspecific (see the text). Bands in the region marked by ** represent Keap1.

We examined whether the Keap1-Nrf2 pathway was involved in the ARE activation induced by 3 mM HEMA, which did not reduce the cellular GSH level (Figure 2B). We thus studied the effect of expression of Nrf2 and Keap1 on the HEMA (3 mM)-induced ARE activation. HepG2 cells were transfected with Nrf2 and/or Keap1 expression vectors in addition to the ARE-luciferase vector pGL4.24-2E, and incubated for 6 h without or with 3 mM HEMA (Figure 3A). Expression of Nrf2 alone significantly increased the basal ARE activity, and the HEMA-induced activation rate of ARE activity in the Nrf2-overexpressing cells (1.3-fold) was less than in the control cells (14-fold). On the other hand, co-expression of Nrf2 and Keap1 gave a basal ARE activity similar to that observed in the control cells; HEMA induced 19-fold activation of ARE activity. Furthermore, expression of Keap1 alone repressed the basal ARE activity and enhanced the HEMA-induced ARE activation (82-fold).

We next examined the transactivation activity of Nrf2 using a GAL4-luciferase reporter assay in HepG2 cells (Figure 3B). In the absence and presence of 3 mM HEMA, expression of the Nrf2 transactivation domain (Nrf2_TAD_, amino-terminal 1-370 amino acids region) fused to GAL4 DNA-binding domain (GAL4_DBD_) showed high GAL4-luciferase activities, whereas GAL4_DBD_ only gave no detectable GAL4- luciferase activities. The poor dependency of GAL4-luciferase activities on 3 mM HEMA (1.9-fold activation) was improved by co-expression of Keap1 (11-fold activation).

When HepG2 cells were incubated for 3 h without or with 3 mM HEMA, HEMA-induced accumulation of Nrf2 was observed in the total cell lysate and in the nuclear extract upon western blot analysis (Figure 3C, left and center panels). A mobility shift of cytosolic Keap1 in SDS-PAGE was also observed after the treatment of cells with 3 mM HEMA (Figure 3C, right panel). The exact mechanism of the mobility shift of Keap1 induced by HEMA is unknown, but the shift seems to be due to chemical modification of Keap1 by HEMA.

In the western blot analysis of HepG2 cell total lysates using an anti-Nrf2 antibody (Figure 3C, left panel), an intensely stained band marked by an asterisk was observed between 2 bands appearing in a HEMA-dependent manner. The molecule reactive with the anti-Nrf2 antibody was mainly in the cytosolic extract (Figure 3C, center panel). We concluded that this intensely stained band was non-specific for the following reasons: 1) in other works using HepG2 cells, several antioxidant-dependent Nrf2 bands are detected upon western blot analysis mainly in nuclear extracts, but not in cytosolic extracts [25], [26]; 2) in our western blot analysis of the total cell lysate of COS-1 cells transfected with the Nrf2 expression vector (Flag-tagged), we observed that only 2 bands corresponding to those indicated by arrows 1 and 2 in the left panel of Figure 3C with 3 kinds of anti-Nrf2 antibodies including the one used in this work (data not shown). Among the 3 anti-Nrf2 antibodies, only the antibody used here could clearly detect endogenous Nrf2 in HepG2 cells. In the blot stained with an anti-Keap1 antibody, two diffused bands were observed (Figure 3C, right panel), as reported by others [26], [27].

### Establishment of stable clones of HepG2 cells transfected with pGL4.24-2E-Neo

**Figure 4:**
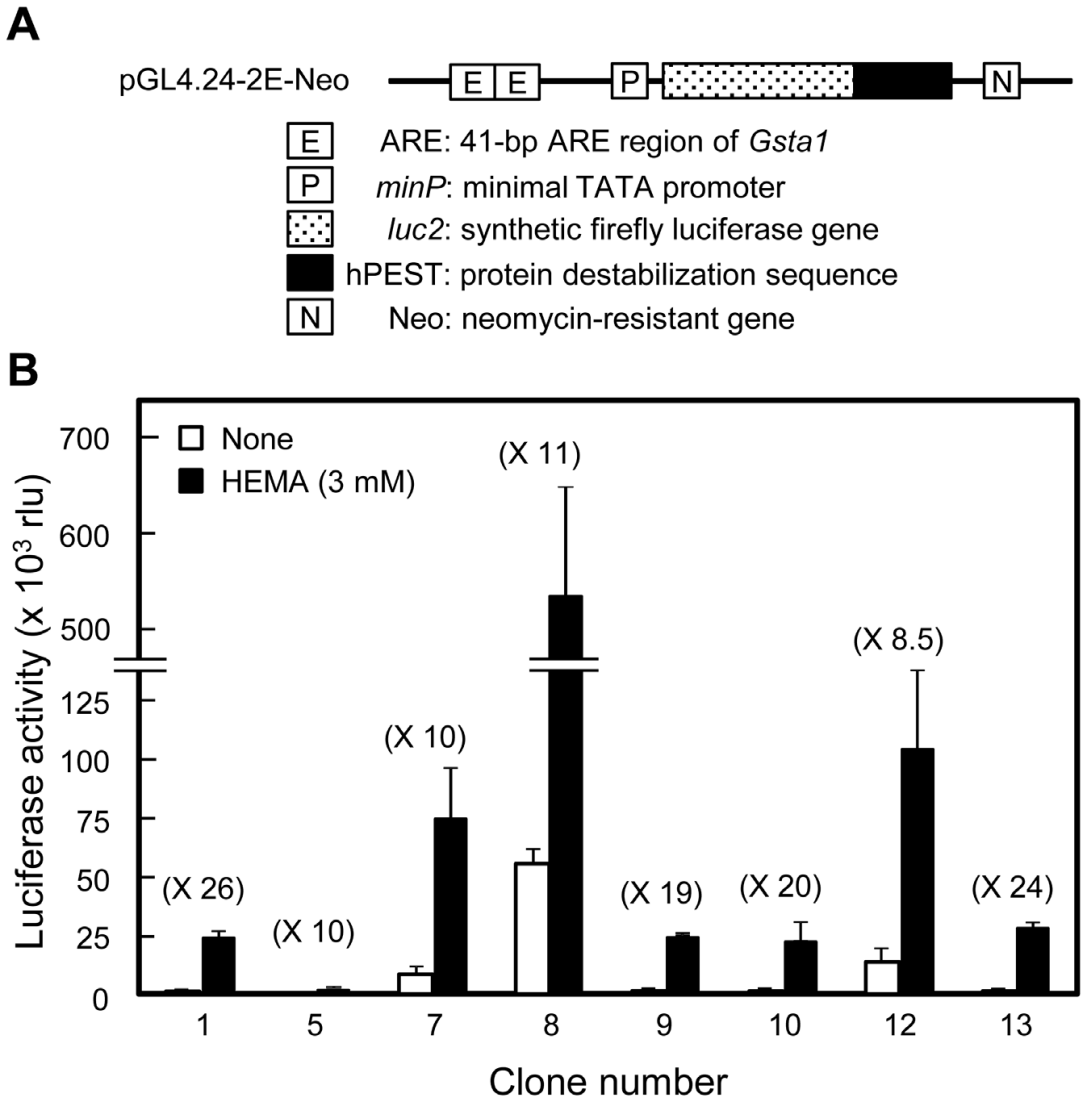
Establishment of stable clones of the HepG2 cell line transfected with the ARE reporter vector pGL4.24-2E-Neo. (A) Structure of reporter construct used for establishing stable clones. The vector pGL4.24-2E-Neo is a variant of pGL4.24-2E containing a neomycin-resistant gene. HepG2 cells stably transfected with pGL4.24-2E-Neo were selected using G418 as described in *Materials and Methods*. (B) Effects of HEMA on ARE-destabilized luciferase reporter activity in stable clonal cells. Selected clones (2 × 10^5^ cells) were cultured in a 24-well plate for 48 h, incubated without or with HEMA (3 mM) for 6 h, and subjected to the assay for the luciferase activities. Data are presented as the mean ± SD (n  =  3–12). Clone numbers 1 and 13 with high response rates were named HepG2-AD1 and HepG2-AD13, respectively.

Stable transfectants were generated by transfection of HepG2 cells with pGL4.24-2E-Neo. G418-resistant clones were isolated and selected with luciferase activities upon exposure to 3 mM HEMA, and 8 clones with significant luciferase activities were obtained (Figure 4A). Among the stable clones, clone numbers 1 and 13 showed high responses to 3 mM HEMA (26-fold and 24-fold, respectively; Figure 4B) and they were named HepG2-AD1 and HepG2-AD13, respectively.

**Figure 5:**
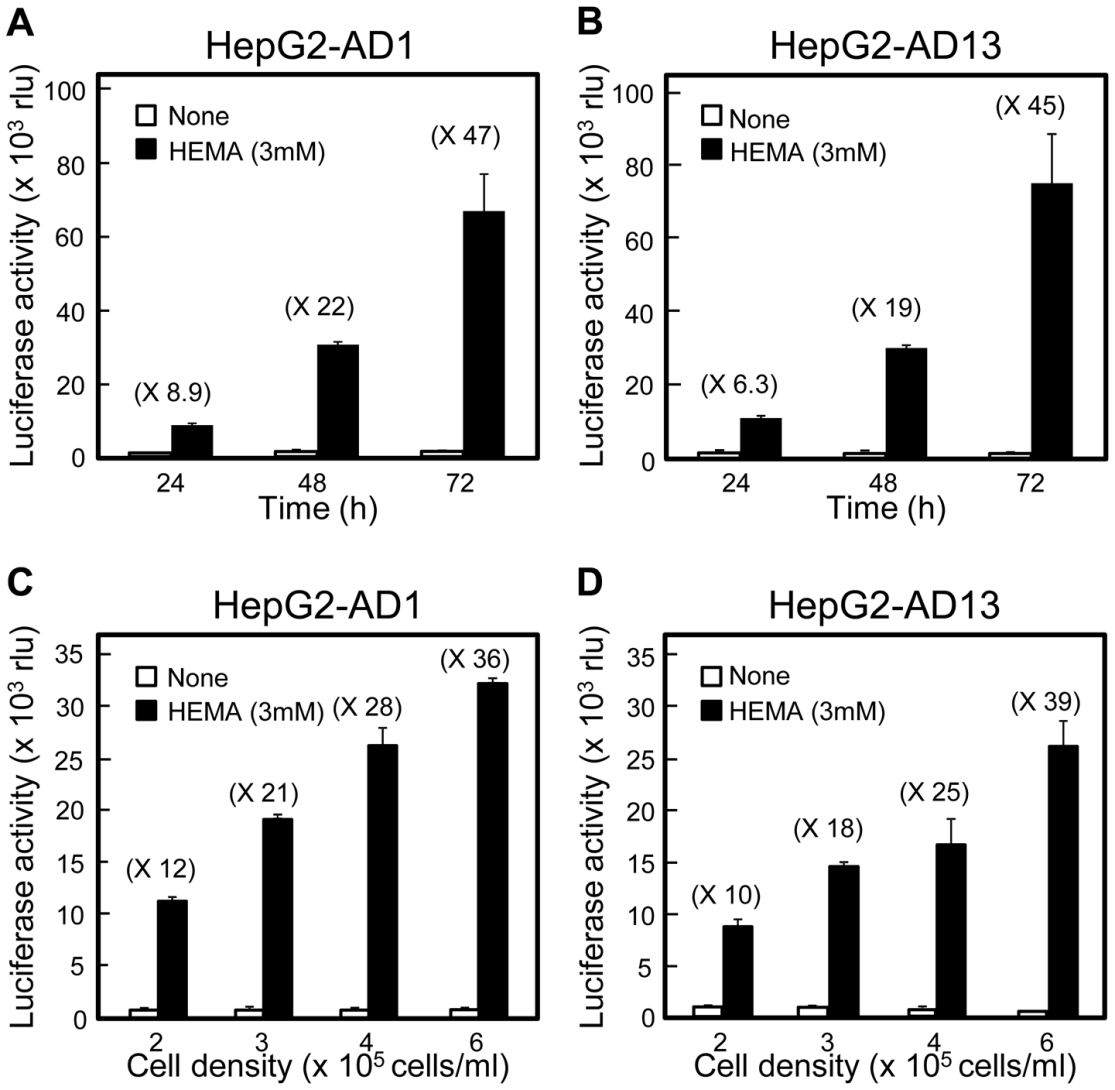
Effect of HepG2-AD1 and HepG2-AD13 cell densities on HEMA-induced ARE activation. (A, B) Culture time-dependent increase in responsiveness to HEMA in HepG2-AD1 and HepG2-AD13 cells. Stable cells (1 × 10^5^ cells) were seeded in a 24-well plate, cultured for 24, 48, or 72 h, incubated for 6 h without or with HEMA (3 mM), and subjected to the assay for luciferase activities. Data are presented as the mean ± SD (n  =  3). (C, D) Cell density-dependent increase in responsiveness to HEMA in HepG2-AD1 and HepG2-AD13 cells. Stable cells (1, 1.5, 2, or 3 × 10^5^ cells) were cultured in a 24-well plate for 24 h, incubated for 6 h without or with HEMA (3 mM), and subjected to the assay for luciferase activities. Data are presented as the mean ± SD (n  =  3). rlu, relative light unit.

The increase in the reporter activity in HepG2-AD1 and -AD13 cells in response to 3 mM HEMA was dependent on culture time between 24 and 72 h (Figure 5A, B) and on cell density between 1 × 10^5^ and 3 × 10^5^ cells/well in a 24-well plate (Figure 5C, D). Thus, the response rate of ARE activity to 3 mM HEMA increased when stable cells were cultured at a high cell density.

### Increased responsiveness of ARE-mediated transcription to resin monomers in the stable cell lines

**Figure 6:**
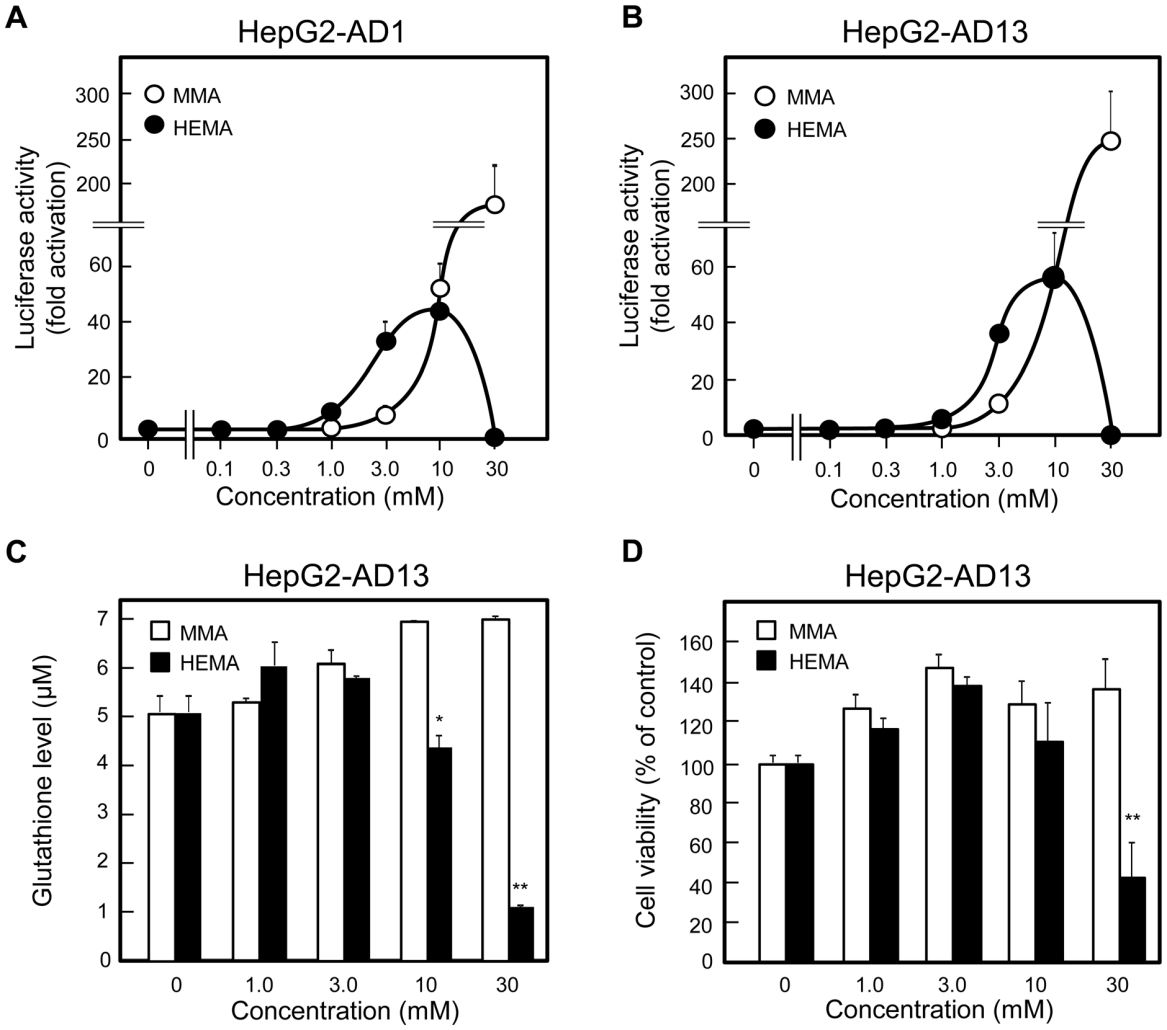
Effect of MMA and HEMA on HepG2-AD1 and HepG2-AD13 cells. (A, B) Effect on promoter activity. HepG2-AD1 (A) and HepG2-AD13 (B) cells (3 × 10^5^ cells) were cultured in a 24-well plate for 24 h, incubated for 6 h with indicated initial concentrations of MMA or HEMA, and subjected to the assay for the luciferase activities, respectively. Data are presented as the mean ± SD (n  =  7–12). (C) Effect on intracellular GSH level. HepG2-AD13 cells were incubated for 6 h with indicated initial concentrations of MMA or HEMA, and subjected to the assay for intracellular levels of GSH. Data are presented as the mean ± SD (n  =  3). (D) Effect on cell viability. Cells were incubated for 24 h with indicated initial concentrations of MMA or HEMA, and subjected to the assay to determine cell viability using WST-8. Cell viability is expressed with the control value, obtained after incubation without exposure to MMA or HEMA, taken as 100%. Data are presented as the mean ± SD (n  =  3). *p < 0.05, **p < 0.01.

HepG2-AD1 and HepG2-AD13 cells (3 × 10^5^ cells) were seeded in a 24-well culture plate for 24 h and incubated without additives or with varying initial concentrations of MMA or HEMA for 6 h. Compared with transiently transfected HepG2 cells, the responsiveness of the reporter gene to MMA and HEMA was significantly improved; in HepG2-AD1 and HepG2-AD13 cells incubated with resin monomers for 6 h, 30 mM MMA increased the reporter activity by up to 171- and 244-fold, respectively; 10 mM HEMA increased it by 47- and 56-fold, respectively, but 30 mM HEMA reduced it below the basal level (0.33- and 0.27-fold, respectively) (Figure 6A, B). Similar to the results observed for transiently transfected cells, HEMA increased the reporter activities at lower concentrations than MMA (Figure 6A, B). In HepG2-AD13 cells, which showed a higher response rate to MMA at 30 mM, HEMA at 10 and 30 mM lowered the intracellular GSH levels after 6 h of incubation (Figure 6C), and 30 mM HEMA lowered cell viability after 24 h of incubation (Figure 6D).

### Dose effects of MMA and HEMA on cellular GSH levels and cell viability in Gin-1 cells

**Figure 7:**
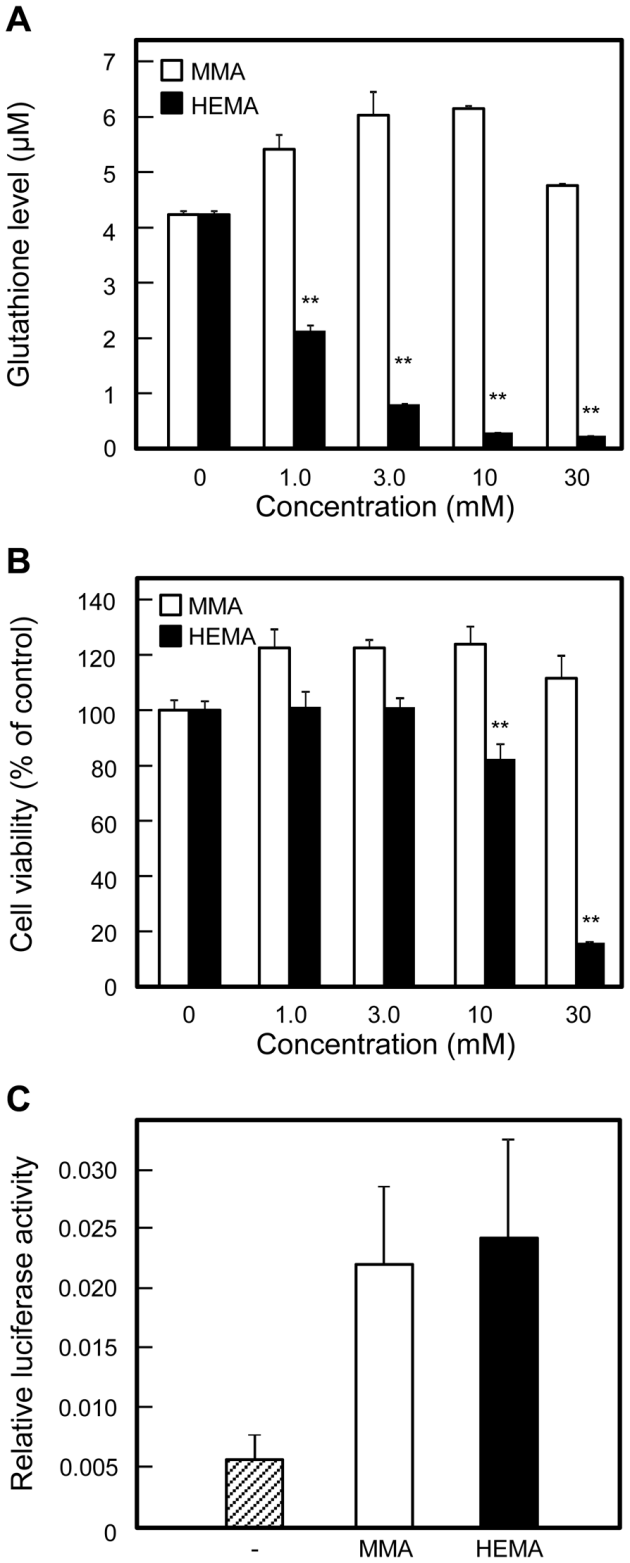
Effect of MMA and HEMA on Gin-1 cells. (A) Effect on intracellular GSH levels. Gin-1 cells were incubated for 6 h without or with indicated initial concentrations of MMA or HEMA, and subjected to the assay for intracellular levels of GSH. Data are presented as the mean ± SD (n  =  3). (B) Effect on cell viability. Gin-1 cells were incubated for 24 h without or with indicated initial concentrations of MMA or HEMA, and subjected to the assay for cell viability using WST-8. Cell viability is expressed with the control value, obtained after incubation without exposure to MMA or HEMA, taken as 100%. Data are presented as the mean ± SD (n  =  3). (C) Effect on promoter activity in Gin-1 cells transfected with the vector pGL4.24-2E. Gin-1 cells were co-transfected with pGL4.24-2E and phRL-CMV, cultured for 24 h, incubated for 6 h without or with MMA or HEMA (10 mM each), and subjected to the assay for the luciferase activities. Relative luciferase activities were expressed with the control value, obtained after incubation without exposure to MMA or HEMA, taken as 1.0. Data are presented as the mean ± SD (n  =  3–8). *p < 0.05, **p < 0.01.

To study dose-dependent effect of MMA and HEMA on oral cells, we used human normal gingival fibroblast Gin-1 cells. When Gin-1 cells were incubated for 6 h with several initial concentrations of MMA and HEMA, they increased and decreased, respectively, the cellular GSH levels in a dose-dependent manner (Figure 7A). HEMA lowered cell viability at 10 and 30 mM after 24-h incubation, but MMA did not (Figure 7B). These results on Gin-1 cells were similar to those on HepG2 cells; however, HEMA reduced the GSH levels and cell viability in Gin-1 cells at lower concentrations than in HepG2 cells.

When Gin-1 cells were transfected with pGL4.24-2E and incubated without additives or with MMA or HEMA (initial concentrations, 10 mM) for 6 h, MMA and HEMA increased the ARE activity 3.7- and 4.0-fold, respectively (Figure 7C).

## Discussion

In this study, we showed that HEMA, a water-soluble resin monomer, induced ARE-mediated transcription. As described above, HEMA is more toxic than MMA and highly increased ARE activity at low concentrations (≤ 3 mM), unlike MMA. It was reported that HEMA is more electronegative than MMA, and that there is a relationship between the high electrophilic reactivity and the high toxicity of dental methacrylates [14]. It was also discussed that the electrophilic reactivity of xenobiotics is closely correlated with the potency of induction of phase II genes and modification of reactive sulfhydryl groups in the sensor protein Keap1 [24]. Therefore, the low-concentration effect of HEMA on ARE activity may be correlated with its high electrophilic reactivity and high cytotoxic potency.

In HepG2 cells transfected transiently and also stably with the ARE-luciferase reporter vectors pGL4.24-2E and pGL4.24-2E-Neo, respectively, HEMA (0.1–3 mM) and MMA (1–30 mM) increased ARE activity, whereas they did not reduce intracellular GSH levels. These results suggest that HEMA and MMA can activate ARE-mediated transcription without accumulation of reactive oxygen species following depletion of GSH levels. In preliminary study, we observed that the activation rate of ARE activity induced by 10 mM HEMA was not decreased by 2-h pretreatment or 6-h coincubation with 10 mM N-acetylcysteine, which is known to reduce oxidative stress-induced, ARE-mediated gene expression [30], [31]. Thus, our data suggest that the ARE activation induced by low concentrations of HEMA and MMA is mainly independent of oxidative stress.

Our expression study on Keap1 and Nrf2 indicated that they were involved in HEMA (3 mM)-induced ARE activation. In addition, our GAL4-reporter assay showed that 3 mM HEMA increased Keap1-dependent Nrf2 transactivation activity. With western blot analysis, we showed that 3 mM HEMA induced a mobility shift of Keap1 and nuclear accumulation of Nrf2. These results suggest that the Keap1-Nrf2 pathway is involved in ARE activation induced by 3 mM HEMA, which did not deplete cellular GSH levels. It is reported that several electrophilic compounds bind cysteine residues located within the intervening region of the sensor protein Keap1 to induce gene expression of phase II enzymes [32]. Taken together, our data support the direct modification of cysteine residues in Keap1 by low concentrations of MMA and HEMA.

In HepG2 cell lines, 30 mM HEMA depleted intracellular GSH levels during 6-h incubation, followed by reduction of cell viability after 24-h incubation, suggesting oxidative stress-dependent cytotoxicity at high concentrations of HEMA. After 6-h incubation with 30 mM HEMA, ARE-dependent promoter activity was below the basal level; SV40, TK, and CMV promoter activities were also substantially reduced. These results suggest nonspecific inhibition of transcription by 6-h incubation with 30 mM HEMA. Similarly to other electrophilic xenobiotics, dental resin monomers can damage DNA, RNA, and protein synthesis [3], [33]. The mechanism of the reduction of ARE activity with 30 mM HEMA is still unknown, but it could be due to nonspecific inhibition of RNA synthesis by undetoxified HEMA.

We established 2 stable HepG2 cell lines (HepG2-AD1 and HepG2-AD13 cells) expressing ARE-destabilized luciferase reporter genes, which showed high response rates to resin monomers. In the stable clones, culture conditions at high cell densities enhanced the response rates to HEMA and MMA. It has been reported that detoxification activity is dependent on the cell density of HepG2 cells and primary cultured hepatocytes [34], [35]. An easy control of cell density for enhancing the response rate to resin monomers is an advantage in using stable cell lines for evaluating ARE-mediated transcriptional activity.

By developing the ARE-luciferase reporter assay system, we showed that HEMA effectively increased ARE enhancer activity at concentrations lower than those required for reduction of cell viabilities. Our data suggest that the low-concentration effect of HEMA on ARE activity reflects its cytotoxicity. Additionally, the incubation time of cells with resin monomers in the ARE-luciferase reporter assay was shorter than those for usual cell viability assays (6 h versus 24 h under our experimental conditions). Thus, our newly developed ARE-luciferase reporter assay system can be used as a rapid and sensitive method for evaluating the cytotoxicity of resin monomers.

As discussed in our previous study [16], the liver is the major organ involved in detoxification of xenobiotics, and hepatic cells, like HepG2 cells, seem to be suitable for the assay of ARE activity. To evaluate whether the data on HepG2 cells were relevant for estimating cytotoxicity of resin monomers in oral cells, we studied dose effects of HEMA and MMA on Gin-1 cells, a normal gingival cell line. The results showed that HEMA, but not MMA, reduced cellular GSH levels and cell viability in Gin-1 cells similarly in HepG2 cells. In Gin-1 cells transfected with the ARE-luciferase vector pGL4.24-2E, the activation rates of ARE activity induced by MMA and HEMA at 10 mM were relatively low, and it was hard to examine their effects on the ARE activity at lower concentrations.

In conclusion, our study suggests that the low-concentration effect of HEMA on ARE activity reflects its cytotoxicity. Our reporter system on ARE activity may be useful for evaluating cytotoxicities of resin monomers at concentrations lower than those required for reduction of cell viabilities. This system may also be useful for determining safe concentrations of resin monomers and for developing dental materials.

## Materials and Methods

### Plasmids

A luciferase reporter vector containing a 5′-flanking region (-990 to +46 bp) of *Gsta1* (ENSMUSG00000074181), pGL4.10-*Gsta1*pro990, was prepared as described previously [16]. The sequence of the 41-bp ARE region [17], [25], [36] of *Gsta1* is as follows: 5′-TAGCTTGGAAATGACATTGCTAATGGTGACAAAGCAACTTT-3′ (-729 to -689 bp; 2 ARE consensus sequences underlined). The 41-bp *Gsta1*-derived ARE was used for the construction of ARE-firefly luciferase reporter vectors as described below.

Vectors carrying 1, 2, or 3 tandem copies of the *Gsta1*-ARE upstream of a minimal TATA promoter (*minP*) in a luciferase vector encoding a synthetic firefly luciferase gene *luc2* (pGL4.23, Promega, Madison, WI) were referred to as pGL4.23-1E, pGL4.23-2E, and pGL4.23-3E, respectively (Figure 1A) and constructed as follows. Sense and antisense oligonucleotides of the *Gsta1*-ARE with *Kpn*I- and *Sac*I-digested sites were phosphorylated by T4 polynucleotide kinase (Takara Bio, Otsu, Japan), annealed to form double-stranded oligonucleotides, and then inserted into the *Kpn*I/*Sac*I sites of pGL4.23 to create pGL4.23-1E. According to the same procedure, the *Gsta1*-ARE oligonucleotide with *Sac*I- and *Nhe*I-digested sites was inserted into *Sac*I/*Nhe*I sites of pGL4.23-1E to create pGL4.23-2E, and then the *Gsta1*-ARE oligonucleotide with *Nhe*I- and *Xho*I-digested sites was inserted into *Nhe*I/*Xho*I sites of pGL4.23-2E to create pGL4.23-3E.

Destabilized luciferase vectors carrying 2 copies of the *Gsta1*-ARE upstream of *minP* in the luciferase vectors pGL4.24 and pGL4.25 (Promega) encoding *luc2P* (firefly luciferase reporter gene fused to a protein degradation sequence hPEST) and *luc2CP* (firefly luciferase reporter gene fused to a protein degradation sequence hCL1 and hPEST), respectively, were prepared as follows. A DNA fragment containing 2 copies of the *Gsta1*-ARE in pGL4.23-2E was digested using *Kpn*I and *Nhe*I restriction enzymes, and then inserted into the *Kpn*I/*Nhe*I sites of pGL4.24 and pGL4.25 to create pGL4.24-2E and pGL4.25-2E, respectively.

A neomycin-resistant ARE-destabilized luciferase vector, pGL4.24-2E-Neo, was prepared as follows. The *Kpn*I/*Eco*RI-digested DNA fragment of pGL4.18 (Promega) containing a neomycin-resistant gene was recombined with the *Kpn*I/*Eco*RI-digested DNA fragment of pGL4.24-2E containing 2 copies of the *Gsta1*-ARE and *minP* to create pGL4.24-2E-Neo (Figure 3A).

A vector to express FLAG (DYKDDDDK)-tagged human Nrf2 at amino-terminus, pFlag-CMV2-hNrf2, was prepared as follows. The *Cla*I/*Xba*I-fragment of human Nrf2 was obtained from IMAGE cDNA clone 4548874 by PCR amplification (25 cycles, 15 sec at 98°C, 15 sec at 55°C, 120 sec at 68°C) using KOD-plus (ver.2)-DNA polymerase with a primer set of hNrf2-P1 (T TCA TCG ATA **ATG** ATG GAC TTG GAG CTG CCG; *Cla*I site underlined, 1st Met of human Nrf2 bolded) and hNrf2-P2 (GCT TCT AGA **TTA** GTT TTT CTT AAC ATC TGG CTT CTT; *Xba*I site underlined, stop codon bolded). The PCR fragment was digested with *Cla*I and *Xba*I, and then inserted into *Cla*I/*Xba*I sites of pFlag-CMV2 (Sigma, St. Louis, MO) to create pFlag-CMV2-hNrf2.

A vector to express human Nrf2 transactivation domain (amino acids 1-370) [37] fused to GAL4 DNA binding domain (amino acids 1-147), pFA-hNrf2, was prepared as follows. The *Bam*HI/*Xba*I-fragment of human Nrf2 transactivation domain was obtained from the IMAGE cDNA clone 4548874 by PCR amplification (25 cycles, 15 sec at 98°C, 15 sec at 55°C, 60 sec at 68°C) using KOD-plus (ver.2)-DNA polymerase with a primer set of hNrf2-P3 (CCG GGA TCC **ATG** ATG GAC TTG GAG CTG CCG; *Bam*HI site underlined, 1st Met of human Nrf2 bolded) and hNrf2-P4 (GCT TCT AGA **TTA** TAG TGT GTC TCC ATA GCT GGA AGA; *Xba*I site underlined, stop codon bolded). The PCR fragment was digested with *Bam*HI and *Xba*I, and then replaced with *Bam*HI /*Xba*I fragment of ATF-2 transactivation domain in pFA-ATF-2 (Stratagene, La Jolla, CA) to create pFA-hNrf2.

For expression of wild type human Keap1, the vector of IMAGE cDNA clone 3910857 (pCMV-SPORT6/hKeap1) was used.

### Cell Culture of HepG2 cells and Gin-1 cells

Human hepatoma HepG2 cells (RIKEN Cell Bank, RCB1648; Tsukuba, Japan) and human normal gingival fibroblast Gin-1 cells (DS Pharma Biomedical, Osaka, Japan) were maintained in Dulbecco’s minimum essential medium (Wako, Osaka, Japan) supplemented with 10% heat-inactivated fetal bovine serum (Equitech-Bio Inc., Kerrville, TX, USA) at 37°C in a 5% CO_2_ incubator. The medium was changed every other day.

### Transient transfection and luciferase assay

HepG2 cells were seeded into 24-well plates (Falcon; Becton Dickinson Labware, Lincoln Park, NJ, USA) at a density of 1.0 × 10^5^ cells/well, cultured for 24 h to 80% confluence, and transfected with ARE-luciferase reporter vectors. A *Renilla* luciferase vector carrying the cytomegalovirus (CMV) promoter, phRL-CMV (Promega), was used as an internal control. The cells were co-transfected with firefly and *Renilla* luciferase reporter vectors (0.5 mg/well) with Lipofectamine LTX (Life Technologies, Carlsbad, CA, USA), according to the manufacturer’s instructions. Transfections were performed in triplicate. Unless otherwise stated, 24 h after transfection, the medium containing MMA (Kanto Chemical, Tokyo, Japan) or HEMA (Kanto Chemical) was added into each well at indicated concentrations; we directly diluted neat HEMA and MMA with the culture medium immediately before use and added the resultant medium to the wells. Cells were cultured for 6 h without changing the medium. Because MMA is volatile, care was taken to avoid interference among wells; we used separate plates for treatment of cells with different concentrations of MMA, and the plates containing MMA were placed in an incubator different from that used for control plates. In this work, we studied the effect of transient exposure of cells to resin monomers, and we did not attempt to maintain a specific concentration of resin monomers in the medium. At the end of culture, the medium was aspirated; the cells were rinsed with phosphate-buffered saline and subjected to lysis using the Passive Lysis Buffer (Promega). The lysates were assayed for firefly and *Renilla* luciferase activities using the Dual-Luciferase Reporter Assay System (Promega) in an AB-2200 luminometer (ATTO, Tokyo, Japan). The firefly luciferase activities were normalized to *Renilla* luciferase activities.

To determine the effect of co-expression of Nrf2 and Keap1, we co-transfected HepG2 cells with the pGL4.24-2E ARE-luciferase vector and pFlag-CMV2-hNrf2 or/and pCMV-SPORT6/hKeap1; pcDNA3 (Life Technologies) was used as a control vector. As an internal control, phRL-CMV was also co-transfected. The luciferase activities were determined as described above.

To examine Nrf2 transactivation activity, we co-transfected HepG2 cells with pFA-Nrf2 and/or pCMV-SPORT6/hKeap1, in addition to a GAL4-responsive luciferase vector, pFR-Luc (Stratagene). pFC-dbd (Stratagene), a vector to express GAL4 DNA binding domain only, and pcDNA3 were used as negative controls for pFA-Nrf2 and pCMV-SPORT6/hKeap1, respectively. As an internal control, phRL-CMV was also co-transfected. The luciferase activities were determined as described above.

### Western blot analysis

Western blot analyses were performed using anti-Nrf2 (EP1808Y, Abcam, Cambridge, UK) and anti-Keap1 (D6B12, Cell Signaling Technology, Beverly, MA) rabbit monoclonal antibodies as follows. For preparation of total cell lysates, HepG2 cells were seeded in 60-mm cell culture dishes (Falcon) at a density of 2 × 10^6^ cells/dish, cultured for 24 h, and treated with resin monomers for 3 h. The cells were washed 3 times with PBS, lysed with 250 µl of 1 × Laemmli’s sample buffer, and sonicated for 10 s with an UP50H ultrasonic processor with 1 mm diameter tip (intensity  =  0.4, Hielscher, Teltow, Germany). For preparation of cytosolic and nuclear extracts, HepG2 cells were seeded in 100-mm cell culture dishes (Falcon) at a density of 4 × 10^6^ cells/dish, cultured for 24 h, and treated with resin monomers for 3 h, and cytosolic and nuclear extracts were prepared with the Nuclear Extract Kit (Active Motif, Carlsbad, CA) according to the manufacturer’s instructions. Protein concentration was determined by the method of Bradford using a protein assay kit (Bio-Rad, Hercules, CA) with bovine serum albumin as a standard. Total cell lysates (10 µl each) and cytosolic and nuclear extracts (10 µg each) were subjected to SDS-PAGE (SuperSep Ace 10% gel, Wako) under reducing conditions. The separated proteins were transferred to a PVDF membrane (Immun-Blot, Bio-Rad), and then the membrane was incubated for 1h with PBS-0.1%Tween20 containing 5% skim milk (Wako). After incubation for 1 h with the primary anti-Nrf2 or anti-Keap1 antibodies (1∶1000 dilution), the membrane was further incubated for 1 h with the secondary HRP-conjugated anti-rabbit IgG antibody (1:5000, GE Healthcare UK Ltd). The proteins on the membrane were visualized using a Luminata Forte Western HRP substrate kit (Millipore) and a LAS-4000mini image analyzer (GE Healthcare).

### GSH measurements

Intracellular GSH levels were quantified using the bioluminescent GSH-Glo glutathione assay (Promega), according to the manufacturer’s instructions. Briefly, HepG2 cells were seeded in a 96-well cell culture plate (Falcon) at a density of 5 × 10^3^ cells/well, incubated for 24 h, and the adherent cells in each well were directly dissolved in 100 µL of GSH-Glo lysis and reaction buffer (Promega). After further addition of 100 µL of GSH-Glo Luciferin detection reagent (Promega) to each well, luminescence was measured using a Mithras LB940 microplate reader (Berthold, Bad Wildbad, Germany). Luminescence values were converted to GSH concentrations based on a standard curve created by serial dilution of a GSH standard according to the manufacturer’s instructions.

### Cell viability assay

Cell viability was measured using the Tetra-Color One assay (Seikagaku Kogyo, Tokyo, Japan), according to the manufacturer’s instructions. Briefly, the cells were seeded in 96-well cell culture plates (Falcon) at a density of 5 × 10^3^ cells/well, cultured for 24 h, and treated with resin monomers in 96-well culture plates, and added with the Tetra-Color One reagent (10 µL to each well) containing tetrazolium salt (WST-8). The plates were incubated for 4 h, and absorbance at 450 nm was measured using the Mithras LB940 microplate reader (Berthold).

### Stable reporter cell lines

To establish the stable transformants of the HepG2 cell line containing the ARE-destabilized luciferase reporter gene, cells were seeded at 4 × 10^5^ cells in a 35-mm dish, cultured for 24 h, and transfected with pGL4.24-2E-Neo (2 mg) using Lipofectamine LTX (Invitrogen). Two days later, cells were trypsinized, and seeded at 3.75 × 10^4^ cells in a 100-mm dish, then grown in the culture medium containing 1 mg/mL G418 (Life Technologies). Individual resistant clones were isolated 3 weeks later and expanded into cell lines. Obtained stable transformants were maintained in minimum essential medium supplemented with 10% heat-inactivated fetal bovine serum and 1 mg/mL G418.

To measure the luciferase activity of the stable transformants, the cells were seeded into 24-well plates at a density of 1–3 × 10^5^ cells/well and cultured for 24–72 h. The medium was replaced with culture medium with or without each resin monomer; the cells were cultured for 6 h, and the luciferase activity was measured using the Luciferase Assay System (Promega) with the AB-2200 luminometer (ATTO).

After screening on luciferase activity in response to 3 mM HEMA, we obtained HepG2-AD1 and HepG2-AD13 cells that were HepG2 cells stably transfected with pGL4.24-2E-Neo. Thus, HepG2-AD1 and HepG2-AD13 cells are unpublished, genetically-modified clones of the HepG2 cell line; however, they are not de novo cell lines established from organs or tissues newly donated by someone.

In our experimental conditions, the luciferase activities derived from the ARE-destabilized luciferase reporter gene in HepG2-AD1 and HepG2-AD13 cells were at least 1000-fold lower than those of the GSH-Glo Luciferin detection reagent; the former luciferase activities gave little effect on the GSH measurement described above.

### Statistical analyses

The data are presented as mean ± standard deviation (SD) of the results obtained from at least 3 separate experiments performed in triplicate. Student’s *t* test was used for statistical evaluation of the effect of the resin monomers. Statistical significance was accepted at a p value of <0.05.

## Supporting Information

Figure S1 **Effect of 30 mM HEMA on several virus-derived promoter activities in HepG2 cells.** (A) Procedure of the luciferase reporter assay using HepG2 cells transiently transfected with several reporter vectors. HepG2 cells (4 × 10^5^ cells) were transiently transfected with one type of 3 reporter vectors (pGL4.13, pGL4.74, and pGL4.75) in a single 35-mm dish. After incubation for 24 h, cells were trypsinized, collected, and divided into each well of a 24-well plate (1 × 10^5^ cells/well). After further incubation for 24 h, cells were incubated for 6 h without or with HEMA (30 mM), and subjected to the assay for luciferase activity. (B) Structures of reporter constructs. The vector pGL4.13 is a plasmid containing the simian virus 40 (SV40) promoter immediately upstream of the synthetic firefly luciferase gene *luc2*. The vector pGL4.74 is a plasmid containing the herpes simplex virus thymidine kinase (TK) promoter immediately upstream of the synthetic *Renilla* luciferase gene hRluc. The vector pGL4.75 is a plasmid containing the cytomegalovirus (CMV) promoter immediately upstream of hRluc. (C) Effect of 30 mM HEMA on virus-derived promoter activities. -, without HEMA. Data are presented as the mean ± SD (n  =  3). *p < 0.05, **p < 0.01. rlu, relative light unit.
(TIF)
