# Supplementary figures and images for: Effect of 2-Hydroxyethyl Methacrylate on Antioxidant Responsive Element-Mediated Transcription: A Possible Indication of Its Cytotoxicity
Source: PLoS One. 2013 Mar 14;8(3):e58907. doi: 10.1371/journal.pone.0058907 (PMC3597541; doi:10.1371/journal.pone.0058907)

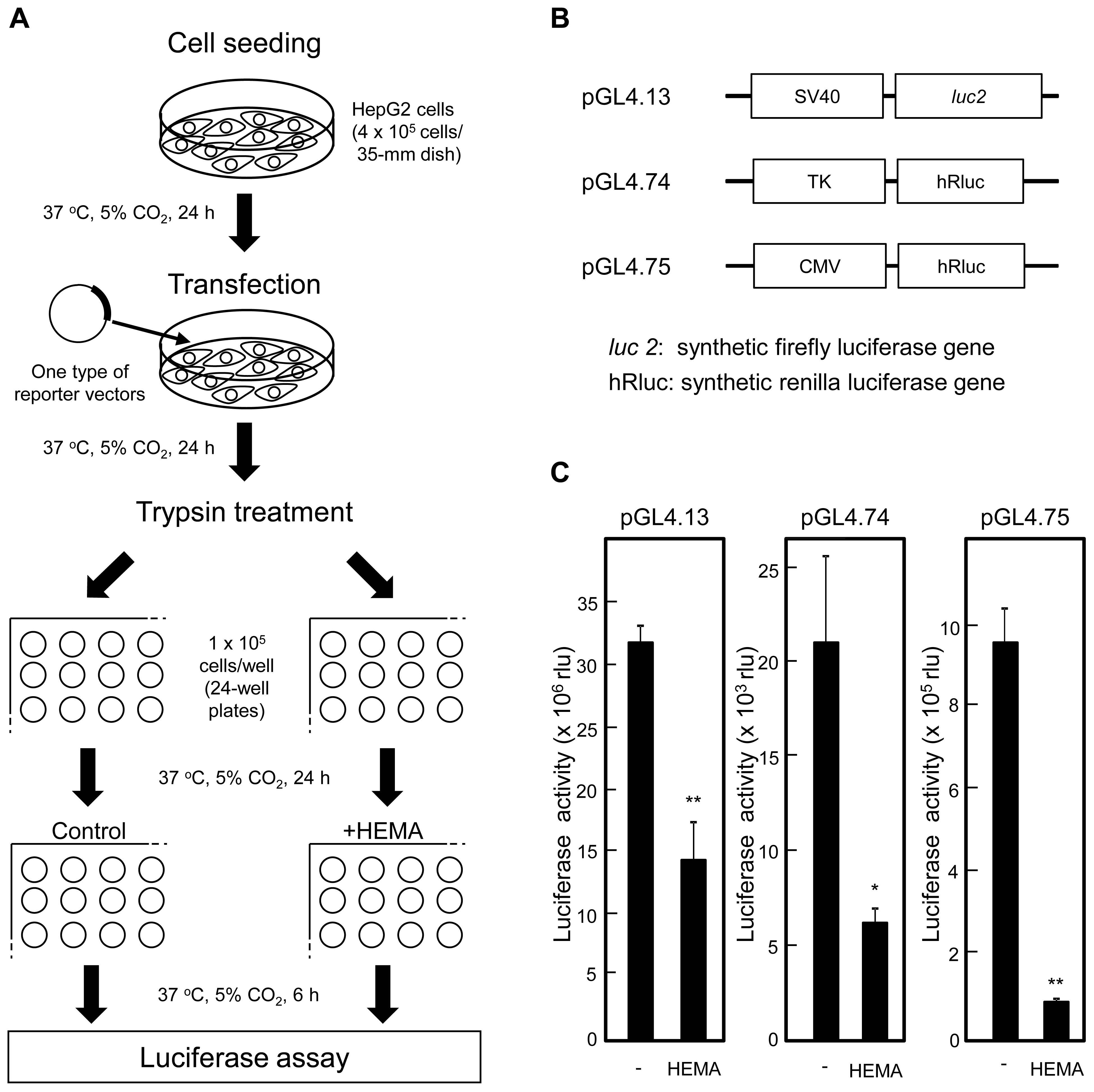

Supplement: Figure S1 — Effect of 30 mM HEMA on several virus-derived promoter activities in HepG2 cells. (A) Procedure of the luciferase reporter assay using HepG2 cells transiently transfected with several reporter vectors. HepG2 cells (4 × 10^5^ cells) were transiently transfected with one type of 3 reporter vectors (pGL4.13, pGL4.74, and pGL4.75) in a single 35-mm dish. After incubation for 24 h, cells were trypsinized, collected, and divided into each well of a 24-well plate (1 × 10^5^ cells/well). After further incubation for 24 h, cells were incubated for 6 h without or with HEMA (30 mM), and subjected to the assay for luciferase activity. (B) Structures of reporter constructs. The vector pGL4.13 is a plasmid containing the simian virus 40 (SV40) promoter immediately upstream of the synthetic firefly luciferase gene luc2. The vector pGL4.74 is a plasmid containing the herpes simplex virus thymidine kinase (TK) promoter immediately upstream of the synthetic Renilla luciferase gene hRluc. The vector pGL4.75 is a plasmid containing the cytomegalovirus (CMV) promoter immediately upstream of hRluc. (C) Effect of 30 mM HEMA on virus-derived promoter activities. -, without HEMA. Data are presented as the mean ± SD (n = 3). *p < 0.05, **p < 0.01. rlu, relative light unit. (TIF) [file pone.0058907.s001.tif]
